# Symptom control in asthma, relationship with life quality and the effect of patient training on the control of symptoms

**DOI:** 10.1186/1939-4551-8-S1-A33

**Published:** 2015-04-08

**Authors:** Nurgül Bozkurt, Ali Ä°Hsan Bozkurt

**Affiliations:** 1 Denizli State Hospital , Turkey; 2 Pamukkale University Faculty of Medicine, Turkey

## Background

The aim of the asthma treatment is symptom control. Symptom control in asthma is affected from a lot of factors. In addition,asthma symptoms affect life quality in a negative way. The relationship between the symptom control level, life quality and spirometric measurements of the patients were investigated. Also the effect of patient education on asthma was investigated.

## Methods

Data were gathered through the questionnaire. Asthma Control Test(ACT) and SF-36 life quality questionnaire(SF-36 LQQ) were applied to the patients. Respiratory functions were measured. Treatments of the patients were given according to GINA guideline. Trainings of patients were offered. Approximately one month later; ACT and SF-36LQQ were repeated.

Data were analyzed through SPSS10.0, and the relationship between ACT and SF-36LQQ was investigated by correlation analysis.

## Results

The mean age of the asthma patients (125) is 46.8 and 77.6% of them is females. 12.8% of the participants have smoking habits, and 54% have allergic rinit, 34.4% have chronic diseases. The initial ACT point mean is 18.4±5.3 and ACT points are similar in both gender(p>0.05).

According to the first ACT, while 13.6% of the patients with asthma had “complete control”, 39.2% had “partly control” on asthma, 47.2% of the patient were “not under control”.

According to the LQQ; Physical Function(PF) point was found as 72.9, Physical Role Restriction(PRR) point as 71.2, Emotional Role Restriction(ERR) point as 70.4, Pain(P) point as 68.9, Social Function(SF) point as 67.9, Vitality(V) point as 58.5, Mental Health(MH) point as 67.7, and Overall Health(OH) point as 62.7.

Significantly positive correlations were found between 4 out of 8 sub categories of life quality (FF,PRR,MH and V) and ACT(p<0.05). However, there is no relationship between ACT and respiration function parameters.

Positive changes in the second ACT were determined. Second ACT point was found as 23.7±2.4 and the difference between two ACT points were found as 5.3. The recovery of ACT are similar in each gender and treatment groups.

A meaningful relationship was found between ACT recovery point and PRR, V points, however, there is no meaningful relationship between the recovery of ACT and respiration function parameters.

## Conclusions

According to the findings,ACT has close relationship with life quality sub-categories in addition to indicating asthma control. For that reason,it would be beneficial to apply in daily practice to reflect an overall evaluation of both the treatments and life quality of the patients.

Our findings show that the trainings to the asthma patients are effective in asthma control.

